# County-Level Structural Racism Indices and Racial Disparities in Lung Cancer Care

**DOI:** 10.1001/jamanetworkopen.2026.13919

**Published:** 2026-05-20

**Authors:** Jacquelyne J. Gaddy, Do H. Lee, Jeph Herrin, James B. Yu, Craig E. Pollack, Lorraine T. Dean, Geoff B. Dougherty, Maureen E. Canavan, Pamela R. Soulos, Cary P. Gross

**Affiliations:** 1 Cancer Outcomes, Public Policy and Effectiveness Research Center, Yale Cancer, Center, New Haven, Connecticut; 2 Department of Internal Medicine, Yale School of Medicine, New Haven, Connecticut; 3 Department of Radiation Oncology, Dartmouth Hitchcock Medical Center, Lebanon, New Hampshire; 4 Department of Health Policy and Management, Johns Hopkins Bloomberg School of Public Health, Johns Hopkins School of Nursing, Baltimore, Maryland; 5 Department of Epidemiology, Johns Hopkins Bloomberg School of Public Health, Baltimore, Maryland; 6 Yale National Clinician Scholars Program, New Haven, Connecticut

## Abstract

**Importance:**

The degree to which structural racism is associated with racial disparities in lung cancer care and outcomes is unknown.

**Objective:**

To evaluate the association between 2 measures of structural racism and quality of care and outcomes for Black and White patients with non–small cell lung cancer (NSCLC) and whether this association differs by patient race.

**Design, Setting, and Participants:**

This cross-sectional study was a retrospective analysis of Medicare beneficiaries diagnosed with NSCLC between 2013 and 2019 using Surveillance, Epidemiology, and End Results Program and Medicare data. Patient inclusion criteria were age older than 67 years at the time of diagnosis, continuous fee-for-service Part A and B Medicare coverage for 24 months before through 12 months after diagnosis, NSCLC histology, and non-Hispanic Black or non-Hispanic White race and ethnicity. The data analysis was performed between September 1, 2024, and April 1, 2025.

**Exposures:**

Two county-level measures of structural racism: the Structural Racism Effect Index, which measures deprivation, and County Structural Racism, which assesses racial dissimilarity between Black and White patients across multiple domains.

**Main Outcomes and Measures:**

The dichotomous outcomes included localized stage at diagnosis, stage-appropriate evaluation and treatment, and 2-year survival. Multivariable mixed-effects logistic regression was used to assess the association between each structural racism measure and outcome, including interaction terms to determine whether structural racism moderated the association between patient race and NSCLC care and outcomes.

**Results:**

Of 54 344 patients (mean [SD] age, 77.7 [6.6] years; 51.6% female), 10.3% were Black and 89.7% were White. A lower proportion of Black patients compared with White patients were diagnosed at a localized stage (30.9% vs 38.4%), received stage-appropriate evaluation and treatment (20.3% vs 28.0%), and survived 2 years after diagnosis (28.7% vs 36.6%). Among White patients, 2-year survival was significantly higher in counties in the highest dissimilarity quintile (estimated probability, 36.8%) vs the lowest (estimated probability, 30.4%), while there was no difference for Black patients (26.8% vs 27.8%). Accordingly, while there was no significant difference in probability of 2-year survival between Black and White patients in the lowest dissimilarity quintile, the disparity was significant in the highest quintile (−10.0%; 95% CI, −12.2% to −7.7%).

**Conclusions and Relevance:**

This cross-sectional study of racial disparities in NSCLC care found that residence in counties with higher structural racism (as measured by the dissimilarity index) was associated with lower mortality among White patients but not among Black patients. Quantifying structural racism and its association with cancer care delivery may identify targets that allow for improvement in quality of cancer care and mitigation of disparities.

## Key Points

**Question:**

Is structural racism associated with quality of care and outcomes for Black and White patients with non–small cell lung cancer (NSCLC)?

**Findings:**

In this cross-sectional study of 54 344 adults with NSCLC, areas with the highest structural racism showed the largest differences in the percentage of Black compared with White patients diagnosed at a localized stage (10.6% lower among Black patients) and who survived 2 years after diagnosis (10.0% lower among Black patients).

**Meaning:**

These findings suggest that quantifying structural racism and its association with NSCLC care delivery may identify targets that allow for improvement in the quality of cancer care and mitigation of racial disparities.

## Teaser

This cross-sectional study evaluates the association between 2 indices of structural racism and quality of care and outcomes for Black and White patients with non–small cell lung cancer at the US county level.

## Introduction

Non–small cell lung cancer (NSCLC) is the most common form of lung cancer and the leading cause of cancer death in the US.^1,2^ While lung cancer incidence and mortality have declined, racial and ethnic disparities have persisted.^2^ Black patients are less likely to be diagnosed with localized disease and to receive appropriate treatment than White patients.^3^ In aggregate, these quality gaps contribute to substantial mortality disparities.^4^ What remains unclear is the contribution of structural racism to these disparities.

Structural racism can be defined as the propagation of discrimination through mutually reinforcing, unjust systems.^5,6^ Overall, there is limited understanding of the association between structural racism and cancer care and outcomes, which may be partly due to the lack of a defined measurement approach. Historically, single measures, such as housing segregation, have been used as a proxy for capturing structural racism within communities. Prior research has also operationalized structural racism as a measure of deprivation, considering multiple area-level measures such as education, employment, poverty, and housing quality.^7^ Socioeconomic disadvantages in communities—or deprivation—are considered a manifestation of these reinforcing, unjust systems. Prior work has found that Black patients with cancer are more likely to live in regions with greater deprivation,^8,9^ which have contributed to racial disparities in lung cancer.^10,11,12,13^ One example of a deprivation metric is the Structural Racism Effect Index (SREI), a regional-level deprivation index that considers 9 domains, including built environment, criminal justice, education, employment, housing, income and poverty, social cohesion, transportation, and wealth.^14^

A limitation of considering deprivation as the sole measure of structural racism is that it does not capture differences between the Black and White populations within communities or regions. To address this concern, measures such as the County Structural Racism (CSR) index have been created. The CSR is a multidomain metric of structural racism that measures dissimilarity (Black compared with White) within counties and across the domains of housing discrimination and segregation, education, access to health care, employment, and criminal justice.^6^ The CSR has been shown to be associated with obesity. Specifically, a higher county-level CSR index value has been associated with a higher body mass index among Black patients but a lower body mass index among White patients.^6^ However, the association between CSR and lung cancer care and outcomes has not been investigated.

In this study, we used the SREI, as a measure of deprivation, and the CSR index, as a measure of dissimilarity, to evaluate the association between structural racism and quality of care for Black and White patients with NSCLC. The CSR was developed to evaluate Black compared with White structural racism due to the long-standing history of systemic inequities experienced by Black people in America.^6^ Given our use of the CSR, the ongoing history of Black individuals facing structural racism, and the substantial number of Black patients in our dataset, we elected to focus on differences between Black and White patients. Moreover, because experiences of structural racism may differentially impact Black and White patients, we examined whether these 2 measures moderated Black-White disparities in care and outcomes. That is, we investigated whether the degree of structural racism within a county changes the association between race and NSCLC care.

## Methods

### Data Source and Study Design

This cross-sectional study used Surveillance, Epidemiology, and End Results (SEER) Program and Medicare data. These data are available through a collaboration between the National Cancer Institute and Centers for Medicare & Medicaid Services. Both county-level measures were linked to SEER-Medicare data on the basis of patient county of residence. The Yale University Institutional Review Board approved this study and deemed it as nonhuman participant research. We followed the Strengthening the Reporting of Observational Studies in Epidemiology (STROBE) reporting guideline for cross-sectional studies.

### Study Sample

We identified Medicare beneficiaries diagnosed with NSCLC between 2013 and 2019. Additional inclusion criteria were continuous fee-for-service Part A and B Medicare coverage for 24 months before through 12 months after diagnosis (or death occurring within 1 year), aged older than 67 years at the time of diagnosis, NSCLC histology, and non-Hispanic Black or non-Hispanic White race and ethnicity. Additional details can be found in the sample construction diagram shown in the eFigure in Supplement 1.

### Outcomes

There were 3 dichotomous outcomes: localized stage at diagnosis (T1-3AN1 vs a higher stage), which was based on National Comprehensive Cancer Network guidelines noting that patients with T1-3AN1 NSCLC may be considered for curative treatment; stage-appropriate evaluation and treatment; and 2-year survival after diagnosis. Stage-appropriate evaluation and treatment was a binary outcome defined as whether a patient received the complete diagnostic evaluation and at least 1 of the recommended treatment modalities for their respective stage based on NCCN guidelines (eTable 1 in Supplement 1).

### Independent Variables

We used 2 different measures to operationalize structural racism (eTable 2 in Supplement 1). The SREI deprivation measure focused on overall wealth and resources in each census tract, incorporating 9 domains of social drivers of health (built environment, criminal justice, education, employment, housing, income and poverty, social cohesion, transportation, and wealth).^14^ The SREI has a mean of 0 and SD of 1. Negative scores reflect areas with increased resources, while positive scores indicate areas with reduced resources. Since the SREI was originally estimated at the census tract level, we aggregated the measure to the county level using weighted averages based on the census tract population. The SREI is publicly available.^15^

The CSR, which focuses on racial dissimilarity within each county, comprises 5 domains (criminal justice, education, employment, housing, and health care)^6^ that are based on either dissimilarity indices or prevalence ratios of the measures for the Black and White populations within a given county. The CSR index was obtained directly from coauthors L.T.D. and G.B.D.

Patient race and ethnicity were identified using Medicare’s Research Triangle Institute race variable, which is based on Social Security Administration data on race that are primarily collected through self-report and an algorithm based on individual name and geography to improve on the identification of Hispanic ethnicity and Asian race. This measure has been validated against patient self-reported race and ethnicity.^16^ We additionally classified patient ethnicity using SEER-provided information, which was abstracted from medical records.

### Covariates

Patient-level sociodemographic variables included socioeconomic status, age, sex, and marital status. For patient-level socioeconomic status, we used dual eligibility for Medicaid and Medicare based on whether patients had at least 1 month of dual eligibility in the year before diagnosis. Neighborhood-level socioeconomic status factors were obtained from the SEER-Medicare database and measured at the census tract level if available; otherwise, they were measured at the zip code level. These factors included median household income and percentage of residents with a high school degree or less. Patient-level clinical factors included cancer stage at diagnosis, comorbidities, receipt of influenza vaccination or primary care encounters in the 2 years prior to diagnosis (proxies for health care access), prior hospitalizations in the year before diagnosis, and frailty. Comorbidity was measured using a variation of the Elixhauser Comorbidity Index, an approach that uses diagnosis codes to identify 30 chronic conditions.^17^ Relevant diagnosis codes were identified from inpatient, outpatient, and physician claims during the 24 through 3 months before cancer diagnosis. Frailty was identified using a validated algorithm that relies on claims in the year prior to diagnosis.^18^

### Statistical Analysis

The data were analyzed between September 1, 2024, and April 1, 2025. We categorized the deprivation and dissimilarity indices into quintiles and calculated the distribution of all covariates across quintiles, estimating the standardized mean difference for each covariate. We examined the crude proportion of patients who experienced each outcome stratified by patient race and quintile of each structural racism index (eTable 3 in Supplement 1). We then used mixed-effects logistic regression, with county-level random effects, to evaluate whether each index was associated with the 3 outcomes. Separate models were run for each measure and outcome. For the outcome of 2-year survival, we did not adjust for stage at diagnosis as our a priori conceptual model included the stage at diagnosis as a mediator of racial differences in survival. Candidate variables with *P* < .20 in bivariate analyses were included as covariates within the multivariable models (eTable 4 in Supplement 1). We then incorporated interaction terms between race and structural racism into each full model to evaluate potential effect modification and assess whether associations differed between racial groups. If the interaction term was statistically significant, we stratified the sample by race and reported adjusted estimated probabilities of the outcome by race and structural racism quintile. Multicollinearity among covariates was evaluated using variance inflation factors, and models were adjusted for statistically significant patient-level characteristics to reduce confounding. Observations with missing data for the primary independent variables (deprivation and dissimilarity index quintiles) were excluded. Missingness among patient-level covariates ranged from less than 0.02% to approximately 8.00%. For these categorical covariates, missing values were assigned to an unknown or missing category. A 2-sided *P* < .05 was the threshold for statistical significance. All statistical analyses were performed using SAS, version 9.4 (SAS Institute Inc); Stata, version 18 (StataCorp LLC); and R, version 4.3.1 (R Foundation for Statistical Computing).

## Results

### Baseline Characteristics of Patients

Our sample included 54 344 individuals, 5588 (10.3%) of whom were non-Hispanic Black compared with 48 756 non-Hispanic White (89.7%) (Table 1). The mean (SD) age was 77.7 (6.6) years, and 51.6% were female and 48.4% male. Of the total sample, 42.3% of patients had stage 4 disease at the time of diagnosis, and 14.8% were dually eligible for Medicare and Medicaid. The distribution of Black patients increased from 6.4% in the lowest deprivation quintile to 15.3% in the highest, with similar findings across dissimilarity quintiles (from 8.2% to 19.2%).

**Table 1.** Sample Characteristics Overall and by County-Level Structural Racism Quintiles

Characteristic	Patients, No. (%)						
	Overall (N = 54 344)	Deprivation measure			Dissimilarity measure		
		Q1 (lowest) (n = 11 020)	Q5 (highest) (n = 10 964)	SMD^a^	Q1 (lowest) (n = 10 886)	Q5 (highest) (n = 11 611)	SMD^a^
Age group, y							
67-69	7047 (13.0)	1264 (11.5)	1686 (15.4)	0.22	1649 (15.1)	1412 (12.2)	0.19
70-74	14 148 (26.0)	2745 (24.9)	3223 (29.4)		3092 (28.4)	2888 (24.9)	
75-79	13 643 (25.1)	2791 (25.3)	2741 (25.0)		2760 (25.4)	2806 (24.2)	
80-84	10 617 (19.5)	2160 (19.6)	1992 (18.2)		2003 (18.4)	2336 (20.1)	
85-94	8889 (16.4)	2060 (18.7)	1322 (12.1)		1382 (12.7)	2169 (18.7)	
Race and ethnicity							
Non-Hispanic Black	5588 (10.3)	701 (6.4)	1682 (15.3)	0.29	894 (8.2)	2234 (19.2)	0.33
Non-Hispanic White	48 756 (89.7)	10 319 (93.6)	9282 (84.7)		9992 (91.8)	9377 (80.8)	
Sex							
Female	28 055 (51.6)	6044 (54.8)	4937 (45.0)	0.20	5148 (47.3)	6192 (53.3)	0.12
Male	>26 278 (>48.4)	>4965 (>45.1)	6027 (55.0)		>5727 (>52.6)	5419 (46.7)	
Unknown or missing	<11 (<0.02)	<11 (<0.10)	0 (0)		<11 (<0.10)	0 (0)	
Marital status							
Married	25 341 (46.6)	5155 (46.8)	5329 (48.6)	0.04	5285 (48.5)	5107 (44.0)	0.10
Unknown or missing	2731 (5.0)	547 (5.0)	558 (5.1)		555 (5.1)	519 (4.5)	
Dual eligibility for Medicaid	8039 (14.8)	1277 (11.6)	2381 (21.7)	0.27	1894 (17.4)	2115 (18.2)	0.02
Household income, mean (SD), $^b^	65 200 (30 500)	86 100 (34 700)	44 600 (16 700)	1.53	52 200 (19 500)	71 300 (37 600)	0.64
% Of region below the federal poverty level, mean (SD)^b^	13.0 (10.5)	7.97 (7.17)	20.1 (10.9)	1.31	16.6 (9.97)	13.1 (12.4)	0.31
% Of region with high school degree or less, mean (SD)^b^	40.5 (17.6)	30.6 (14.5)	54.2 (15.1)	1.60	47.1 (16.6)	36.5 (18.1)	0.61
Stage at diagnosis							
1	13 849 (25.5)	3120 (28.3)	2424 (22.1)	0.15	2606 (23.9)	2932 (25.3)	0.11
2	4490 (8.3)	887 (8.0)	978 (8.9)		902 (8.3)	888 (7.6)	
3	9491 (17.5)	1806 (16.4)	2073 (18.9)		1965 (18.1)	1927 (16.6)	
4	22 995 (42.3)	4549 (41.3)	4782 (43.6)		4567 (42.0)	5213 (44.9)	
Unknown or missing	3519 (6.5)	658 (6.0)	707 (6.4)		846 (7.8)	651 (5.6)	
Elixhauser Comorbidity Index							
0	13 157 (24.2)	2749 (24.9)	2631 (24.0)	0.03	2792 (25.6)	2711 (23.3)	0.08
1-2	20 047 (36.9)	4146 (37.6)	4098 (37.4)		4075 (37.4)	4182 (36.0)	
≥3	21 140 (38.9)	4125 (37.4)	4235 (38.6)		4019 (36.9)	4718 (40.6)	
Receipt of influenza vaccination	37 598 (69.2)	8068 (73.2)	6947 (63.4)	0.21	7208 (66.2)	7936 (68.3)	0.05
Prior hospitalizations	14 352 (26.4)	2882 (26.2)	3005 (27.4)	0.03	2838 (26.1)	3310 (28.5)	0.06
Primary care practitioner visit	45 180 (83.1)	9381 (85.1)	8515 (77.7)	0.19	8613 (79.1)	9723 (83.7)	0.12
Frailty							
Not frail	1701 (3.1)	422 (3.8)	285 (2.6)	0.11	334 (3.1)	348 (3.0)	0.05
Prefrail	31 853 (58.6)	6658 (60.4)	6286 (57.3)		6389 (58.7)	6609 (56.9)	
Mildly frail	16 060 (29.6)	3075 (27.9)	3374 (30.8)		3205 (29.4)	3506 (30.2)	
Moderately frail	3878 (7.1)	701 (6.4)	840 (7.7)		797 (7.3)	917 (7.9)	
Severely frail	852 (1.6)	164 (1.5)	179 (1.6)		161 (1.5)	231 (2.0)	

Abbreviation: Q, quintile.

^a^ Calculated between Q1 and Q5 of the structural racism measure.

^b^ Calculations exclude missing values.

As expected, patients residing in counties with the highest quintile of deprivation had higher rates of poverty and lower median income than patients in lower quintiles (mean [SD], 20.1 [10.9] in quintile 5 vs 8.0 [7.2] in quintile 1; standardized mean difference, 1.31). Conversely, there was no significant difference in the percentage of the population living below the federal poverty level across dissimilarity quintiles (mean [SD], 16.6% [10.0%] in quintile 1 vs 13.1% [12.4%] in quintile 5; standardized mean difference, 0.31). None of the other clinical factors showed a significant difference across structural racism quintiles (Table 1).

### Association of Race and Structural Racism With Clinical Outcomes

Black patients compared with White patients had significantly lower rates of each outcome (localized stage at diagnosis, 30.9% vs 38.4%; receipt of stage-appropriate evaluation and treatment, 20.3% vs 28.0%; 2-year survival, 28.7% vs 36.6%) (all *P* < .001) (Table 2). There was an inverse association between quintile of deprivation and quality of care. For example, within the lowest quintile of deprivation, 40.5% of patients survived 2 years after diagnosis compared with 30.1% in the highest quintile (*P* < .001). While the dissimilarity quintile was not associated with localized stage at diagnosis, there was a stepwise increase in quality of care in terms of appropriate evaluation and treatment and survival with increasing quintile (Table 2).

**Table 2.** Proportion of Patients With Each Outcome Overall, by Race, and by County-Level Structural Racism

Variable	Patients, No. (%)^a^				Deprivation measure						Dissimilarity measure					
	Overall	Non-Hispanic Black	Non-Hispanic White	*P* value^b^	Patients, No. (%)^a^					*P* value^c^	Patients, No. (%)^a^					*P* value^c^
					Q1 (lowest)	Q2	Q3	Q4	Q5 (highest)		Q1 (lowest)	Q2	Q3	Q4	Q5 (highest)	
No. of patients	54 344	5588	48 756		11 020	10 804	11 276	10 280	10 964		10 886	10 867	10 841	10 139	11 611	
Localized stage at diagnosis	20 443 (37.6)	1724 (30.9)	18 719 (38.4)	<.001	4400 (39.9)	4181 (38.7)	4253 (37.7)	3732 (36.3)	3877 (35.4)	<.001	3949 (36.3)	4114 (37.9)	4126 (38.1)	4021 (39.7)	4233 (36.5)	<.001
Stage-appropriate evaluation and treatment	14 807 (27.2)	1136 (20.3)	13 671 (28.0)	<.001	3395 (30.8)	3292 (30.5)	3068 (27.2)	2604 (25.3)	2448 (22.3)	<.001	2681 (24.6)	2876 (26.5)	3063 (28.3)	2991 (29.5)	3196 (27.5)	<.001
2-y Survival	19 469 (35.8)	1602 (28.7)	17 867 (36.6)	<.001	4467 (40.5)	4149 (38.4)	4116 (36.5)	3439 (33.5)	3298 (30.1)	<.001	3546 (32.6)	3871 (35.6)	3968 (36.6)	3896 (38.4)	4188 (36.1)	<.001

Abbreviation: Q, quintile.

^a^ Percentage calculation based on column percentages, in which the total (denominator) represents the number of patients in each respective quintile of either deprivation (assessed using the deprivation measure or dissimilarity measure).

^b^ Bivariate association (χ^2^ test of proportions) between proportions of Black and White patients with respective outcomes.

^c^ Bivariate association (χ^2^ test) between the specified structural racism metric (deprivation or dissimilarity) and respective patient outcomes.

**Figure 1.**
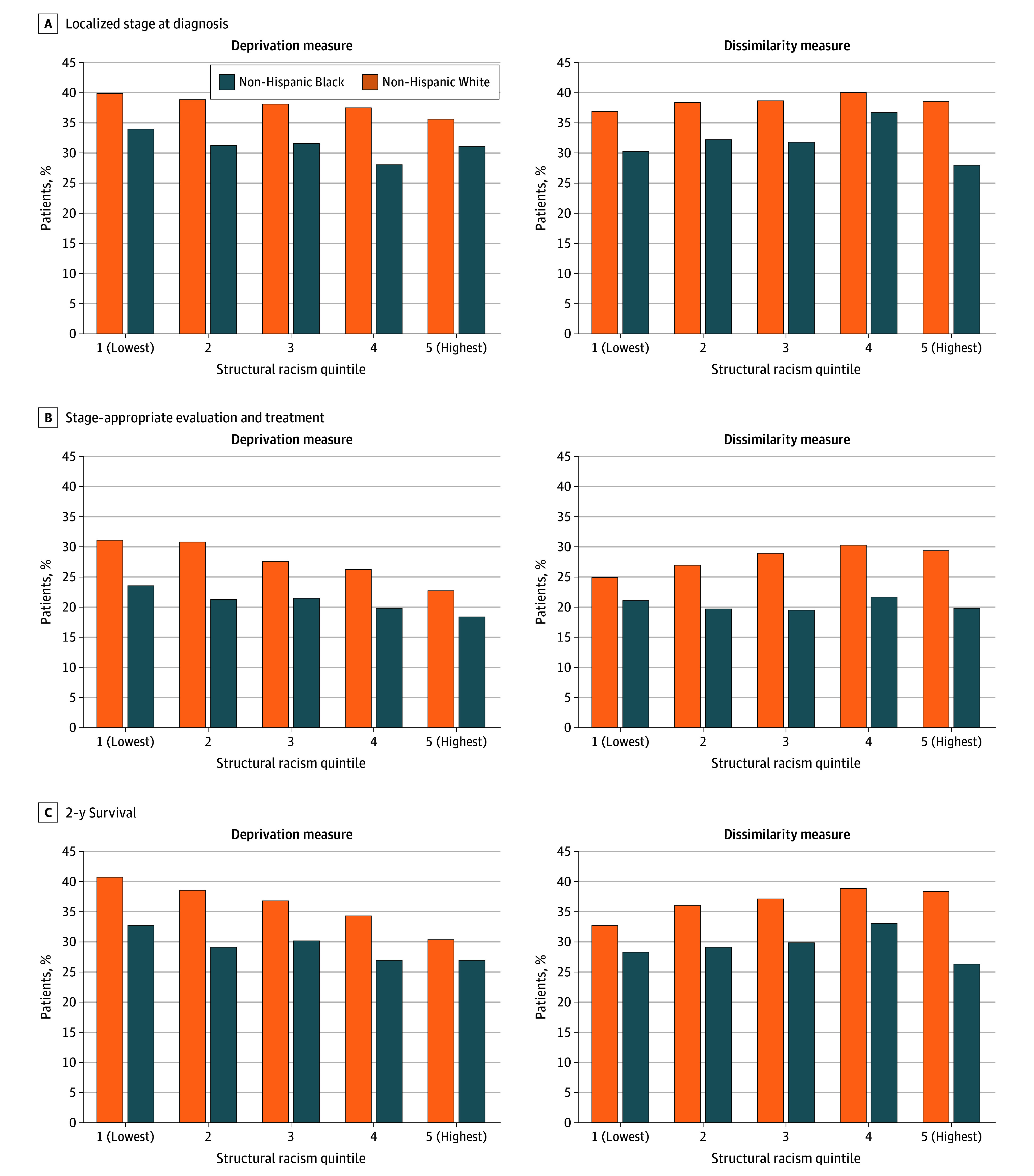
Bar Graph of the Proportion of Patients Who Experienced Each Outcome Stratified by Patient Race and Degree of County-Level Structural Racism

Black patients experienced worse NSCLC care and outcomes compared with White patients across all quintiles of deprivation (Figure 1). However, as deprivation increased, both Black and White patients had poorer outcomes. This pattern was not observed for dissimilarity. As the level of dissimilarity increased, there was no distinct pattern for outcomes among either Black or White patients.

In the adjusted analyses, there was a significant association between county-level deprivation and each of the outcomes (Table 3). Compared with patients living in counties with the lowest deprivation (quintile 1), patients living in counties with the highest (quintile 5) had a lower odds of being diagnosed with a localized stage (adjusted odds ratio [AOR], 0.84; 95% CI, 0.74-0.94) (eTable 5 in Supplement 1), receiving appropriate evaluation and treatment (AOR, 0.69; 95% CI, 0.59-0.80) (eTable 6 in Supplement 1), and surviving 2 years after diagnosis (AOR, 0.66; 95% CI, 0.58-0.75) (eTable 7 in Supplement 1). Additionally, Black patients had a lower odds of being diagnosed with a localized stage (AOR, 0.73; 95% CI, 0.68-0.78), receiving appropriate evaluation and treatment (AOR, 0.71; 95% CI, 0.65-0.76), and surviving 2 years after diagnosis (AOR, 0.76; 95% CI, 0.70-0.81) compared with White patients. Interaction terms between quintile of county deprivation and patient race were not statistically significant for any of the outcomes, indicating that there were no differences in the association between race and quality of care across county deprivation strata (Table 3).

**Table 3.** Adjusted Association Among Patient Race, County-Level Structural Racism, and Each Outcome

Outcome	Deprivation measure^a^			Dissimilarity measure^a^		
	AOR (95% CI)	*P* value		AOR (95% CI)	*P* value	
		Wald	Interaction		Wald	Interaction
**Localized stage at diagnosis**						
Race						
Non-Hispanic Black	0.73 (0.68-0.78)	<.001	.25	0.74 (0.70-0.79)	<.001	.01
Non-Hispanic White	1 [Reference]			1 [Reference]		
Structural racism index quintile						
1 (Lowest)	1 [Reference]	.02	NA	1 [Reference]	.007	NA
2	0.91 (0.79-1.06)			1.02 (0.90-1.15)		
3	0.92 (0.80-1.05)			1.01 (0.89-1.14)		
4	0.84 (0.74-0.96)			1.21 (1.07-1.36)		
5 (Highest)	0.84 (0.74-0.94)			0.96 (0.86-1.08)		
**Appropriate evaluation and treatment**						
Race						
Non-Hispanic Black	0.71 (0.65-0.76)	<.001	.71	0.71 (0.66-0.77)	0.001	.12
Non-Hispanic White	1 [Reference]			1 [Reference]		
Structural racism index quintile						
1 (Lowest)	1 [Reference]	<.001	NA	1 [Reference]	.47	NA
2	0.95 (0.78-1.15)			0.98 (0.83-1.15)		
3	0.87 (0.73-1.04)			0.95 (0.81-1.12)		
4	0.80 (0.68-0.95)			1.10 (0.92-1.30)		
5 (Highest)	0.69 (0.59-0.80)			1.09 (0.92-1.30)		
**2-y Survival**						
Race						
Non-Hispanic Black	0.76 (0.70-0.81)	<.001	.20	0.78 (0.73-0.84)	<.001	.002
Non-Hispanic White	1 [Reference]			1 [Reference]		
Structural racism index quintile						
1 (Lowest)	1 [Reference]	<.001	NA	1 [Reference]	.007	NA
2	0.86 (0.73-1.01)			1.01 (0.88-1.16)		
3	0.81 (0.70-0.94)			1.09 (0.95-1.25)		
4	0.74 (0.65-0.85)			1.29 (1.12-1.49)		
5 (Highest)	0.66 (0.58-0.75)			1.13 (0.98-1.30)		

Abbreviations: AOR, adjusted odds ratio; NA, not applicable.

^a^ Model adjusted for the respective structural racism index, race, age group, sex, marital status, year of diagnosis, comorbidity, hospitalizations in the prior year, and frailty.

Conversely, there was no consistent association between county-level dissimilarity and the 3 outcomes. While there was a significant difference between quintiles 1 and 4 of the dissimilarity index and localized stage at diagnosis (AOR, 1.21; 95% CI, 1.07-1.36) and 2-year survival (AOR, 1.29; 95% CI, 1.12-1.49), there was no pattern between the dissimilarity index and receipt of appropriate evaluation and treatment. The associations between Black race and each outcome were consistent with those from the model adjusting for deprivation (Table 3).

**Figure 2.**
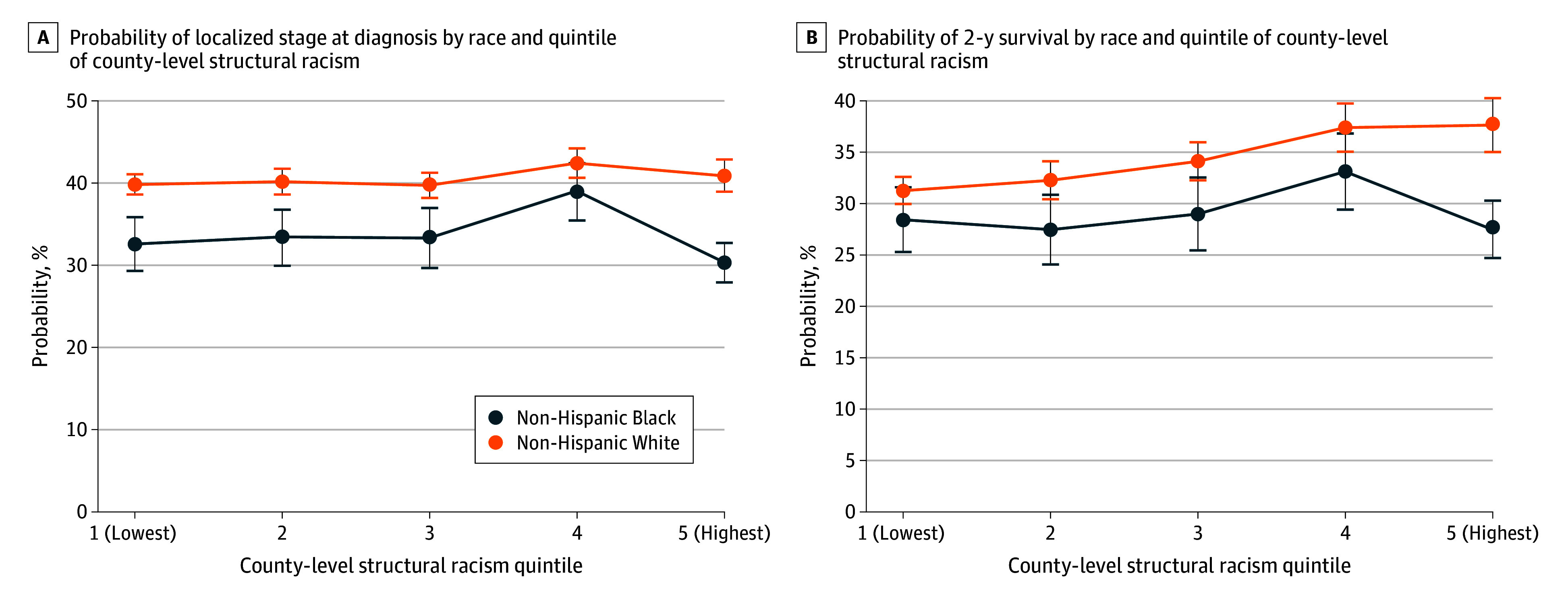
Line Graph of the Estimated Probability of Localized Stage at Diagnosis and 2-Year Survival by Race and Quintile of County-Level Structural Racism Error bars indicate the 95% CI.

There was a significant interaction between patient race and dissimilarity quintile for 2 outcomes, localized stage at diagnosis (interaction *P* = .01) and 2-year survival (interaction *P* = .002). Thus, we calculated the estimated probability of these 2 outcomes stratified by patient race and dissimilarity quintile. Within each racial group, there was no significant difference between dissimilarity quintile and early stage at diagnosis. For instance, among White patients, 39.9% of those in the lowest dissimilarity quintile were diagnosed at an early stage compared with 41.0% of patients in the highest quintile (*P* = .36). Similarly, among Black patients, the likelihood of early-stage diagnosis was 32.6% in the lowest quintile and 30.4% in the highest quintile (*P* = .29). However, the magnitude of disparities between Black and White patients varied across dissimilarity quintiles (Figure 2A).

The disparity for 2-year survival between Black and White patients also varied across quintiles of the dissimilarity index. As structural racism increased, White patients had increased 2-year survival (increasing from 30.4% to 36.8% from the 1st to 5th quintile) (*P* ≤ .001), while Black patients’ probability of survival did not consistently increase. In areas with the lowest dissimilarity (quintile 1), there was no significant difference in 2-year survival between Black and White patients (−2.6%; 95% CI, −5.8% to 0.6%; *P* = .11). Conversely, in the highest dissimilarity quintile, Black patients had an estimated probability of 2-year survival of 26.8% (95% CI, 24.1%-29.5%) compared with an estimated probability of 36.8% (95% CI, 34.2%-39.3%) among White patients, a significant difference of −10.0% (−12.2% to −7.7%) (*P* ≤ .001) (Figure 2B).

## Discussion

This cross-sectional study found associations of disparities between Black and White patients with NSCLC care quality and outcomes that were moderated by county-level structural racism. As expected, we found that Black patients were more likely to live in regions with increased deprivation and that deprivation was associated with the care of both Black and White patients. However, we show that higher structural racism is associated with larger disparities in outcomes. White patients actually tended to have better survival in areas with more structural racism (as measured by the dissimilarity index) compared with White patients in areas with lower structural racism. These racial differences in survival may have been influenced by stage at diagnosis, as White patients had a higher rate of localized stage at diagnosis compared with Black patients. Together, these findings show that heightened structural racism is associated with poorer clinical care for Black patients but not White patients.

These findings show that characterizing regions based on deprivation alone is insufficient when attempting to capture the full association of systemic structural racism with cancer care. While consideration of deprivation allows for the evaluation of area-level social advantage, it does not allow for the evaluation of Black and White population differences.

Prior structural racism studies have focused on residential segregation as opposed to considering the multidomain composition of structural racism. For example, through the use of the Index of Dissimilarity, a validated measure of residential segregation, Munir et al^19^ found that compared with White patients, Black patients with hepatopancreatic biliary cancer living in highly segregated areas were less likely to present with early-stage disease or undergo curative intent resection and had higher mortality. Similarly, several prior studies have shown that residential segregation, as measured by the Index of Concentration at the Extremes, is associated with worse cancer outcomes, including stage at diagnosis for colorectal cancer,^20^ use of appropriate imaging for prostate cancer,^21^ and breast cancer survival.^22,23^ While we did not evaluate residential segregation individually, the dissimilarity index incorporates a measure for the housing inequity domain. In addition to considering the influence of residential segregation on structural racism, this study expands prior literature through the consideration of additional domains such as education, criminal justice, and health care. The use of the Index of Dissimilarity, Index of Concentration at the Extremes, CSR, and other measures shows the growing need to establish a methodological approach to evaluating structural racism and its association with cancer care outcomes.^24^

### Limitations

Our study had several limitations. First, we did not consider patients of other marginalized racial or ethnic backgrounds, such as Asian American, Hispanic, and Pacific Islander. These minoritized groups also experience substantial disparities in NSCLC outcomes and would benefit from the evaluation of the association of structural racism with these established disparities.^3^ Although we explained our rationale for focusing on Black and White patient differences, these methods could (and should) be expanded to other marginalized communities. Second, by using county-level data, we were unable to consider granular details of communities, such as access to fresh foods, crime, walkability, or redlining, all of which may be associated with health disparities.^25^ Third, identification of stage-appropriate treatment using claims data without additional clinical information may have been incomplete or inaccurate. Fourth, patients aged 67 years or younger, those enrolled in Medicare Advantage, and uninsured patients were not represented in this study, although they may also be impacted by structural racism. While the absence of these populations may limit the generalizability of our findings, we believe that the association between structural racism and lung cancer outcomes would not be systematically different in younger patients or patients with other types of insurance. Finally, we were unable to incorporate smoking history, as Medicare claims do not reliably capture this information.^26^ Future work should include tobacco use in the assessment of lung cancer care quality, given that Black communities have been targeted for advertising from tobacco companies through tailored images and other media formats to further attract use,^27^ along with inequitable tobacco retailer availability in marginalized communities.^28^

## Conclusions

This cross-sectional study found substantial county-level gaps in cancer care and outcomes associated with Black and White race among patients diagnosed with NSCLC. Furthermore, the amount of racial inequity within counties had a differential association with survival for Black patients compared with White patients. This finding builds on prior work suggesting that increased structural racism most often correlates with heightened barriers for Black patients but not White patients.^29^ These heightened barriers may reduce access to equitable health care and may ultimately lead to delayed diagnosis and poorer survival for Black patients. By quantifying structural racism and its association with cancer care delivery, we could identify targets that allow for the improvement of the quality of cancer care and mitigation of disparities.

## Notes

Supplement 1. **eFigure. **Sample Construction Diagram **eTable 1.** Stage-Appropriate Evaluation and Treatment Based on National Comprehensive Cancer Network (NCCN) Guidelines **eTable 2.** County-Level Deprivation and Structural Racism Indices Comparison **eTable 3. **Distribution of Patient Race and Outcomes Across Quintiles of Structural Racism Indices **eTable 4.** Association Between Covariates and Outcomes (Bivariate Analysis) **eTable 5.** Association Between Localized Stage at Diagnosis and County-Level Structural Racism **eTable 6. **Association Between Appropriate Evaluation and Treatment and County-Level Structural Racism **eTable 7.** Association Between 2-Year Survival and County-Level Structural Racism

Supplement 2.
Data Sharing Statement
