# Supplementary material for: County-Level Structural Racism Indices and Racial Disparities in Lung Cancer Care
Source: JAMA Netw Open. 2026 May 20;9(5):e2613919. doi: 10.1001/jamanetworkopen.2026.13919 (PMC13191378; doi:10.1001/jamanetworkopen.2026.13919)
Supplement: Supplement 1. — eFigure. Sample Construction Diagram eTable 1. Stage-Appropriate Evaluation and Treatment Based on National Comprehensive Cancer Network (NCCN) Guidelines eTable 2. County-Level Deprivation and Structural Racism Indices Comparison eTable 3. Distribution of Patient Race and Outcomes Across Quintiles of Structural Racism Indices eTable 4. Association Between Covariates and Outcomes (Bivariate Analysis) eTable 5. Association Between Localized Stage at Diagnosis and County-Level Structural Racism eTable 6. Association Between Appropriate Evaluation and Treatment and County-Level Structural Racism eTable 7. Association Between 2-Year Survival and County-Level Structural Racism [file jamanetwopen-e2613919-s001.pdf]

Supplemental Online Content

Gaddy JJ, Lee DH, Herrin J, et al. County-level structural racism indices and racial disparities in lung cancer. *JAMA Netw Open*. 2026;9(5):e2613919. doi:10.1001/jamanetworkopen.2026.13919

eFigure. Sample Construction Diagram

eTable 1. Stage-Appropriate Evaluation and Treatment Based on National Comprehensive Cancer Network (NCCN) Guidelines

eTable 2. County-Level Deprivation and Structural Racism Indices Comparison

eTable 3. Distribution of Patient Race and Outcomes Across Quintiles of Structural Racism Indices

eTable 4. Association Between Covariates and Outcomes (Bivariate Analysis)

eTable 5. Association Between Localized Stage at Diagnosis and County-Level Structural Racism

eTable 6. Association Between Appropriate Evaluation and Treatment and County-Level Structural Racism

eTable 7. Association Between 2-Year Survival and County-Level Structural Racism

This supplemental material has been provided by the authors to give readers additional information about their work.

© 2026 Gaddy JJ et al. *JAMA Network Open*.

eFigure. Sample Construction Diagram

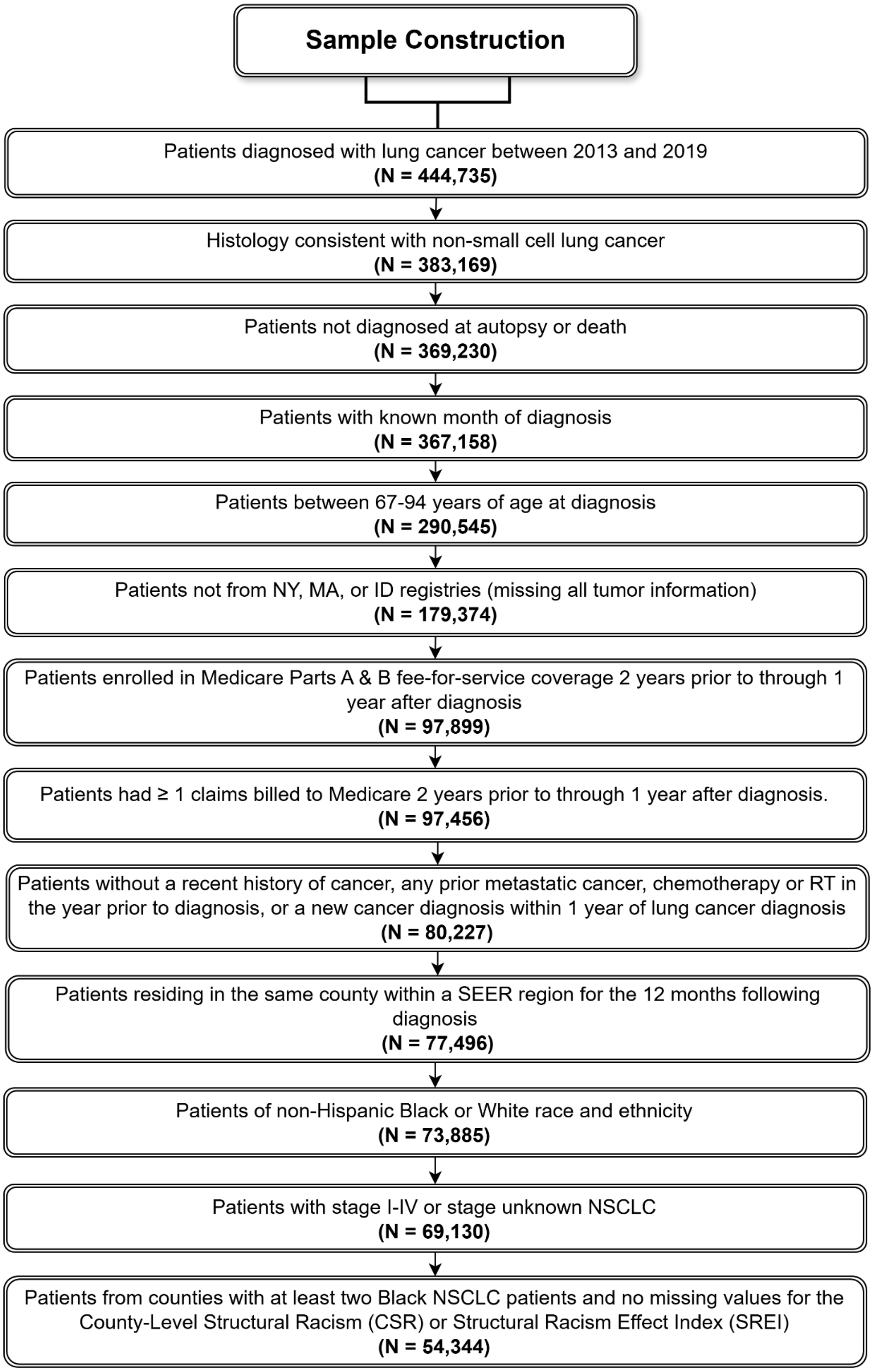

Abbreviations:NY, New York; MA, Massachusetts; ID, Indiana; RT, Radiotherapy; SEER, Surveillance, Epidemiology, and End Results; NSCLC: Non-Small Cell Lung Cancer

© 2026 Gaddy JJ et al. *JAMA Network Open*.

eTable 1. Stage-Appropriate Evaluation and Treatment Based on National Comprehensive Cancer Network (NCCN) Guidelines

eTable 1a. Stage-appropriate evaluation based on NCCN guidelines.

	2013-2016	2017 v3
Stage IA	PFT (if not previously done)<br>Bronchoscopy<br>Pathologic mediastinal lymph node evaluation (category 2B; mediastinoscopy, mediastinotomy, EBUS, EUS, and CT-guided biopsy.)<br>PET/CT scan (if not previously done)	PFT (if not previously done)<br>Bronchoscopy<br>FDG PET/CT scan (if not previously done)
Stage IB	PFT (if not previously done)<br>Bronchoscopy<br>Pathologic mediastinal lymph node evaluation (mediastinoscopy, mediastinotomy, EBUS, EUS, and CT-guided biopsy.)<br>PET/CT scan (if not previously done)<br>Brain MRI	PFT (if not previously done)<br>Bronchoscopy<br>Pathologic mediastinal lymph node evaluation (mediastinoscopy, mediastinotomy, EBUS, EUS, and CT-guided biopsy.)<br>FDG PET/CT scan (if not previously done)
Stage II<br>Stage IIIA	PFT (if not previously done)<br>Bronchoscopy<br>Pathologic mediastinal lymph node evaluation (mediastinoscopy, mediastinotomy, EBUS, EUS, and CT-guided biopsy.)<br>PET/CT scan (if not previously done)<br>Brain MRI	
Stage IIIB (T1-3, N3)	PFT (if not previously done)<br>PET/CT scan (if not previously done)<br>Brain MRI<br>Pathologic confirmation (mediastinoscopy, supraclavicular lymph node biopsy; thoracoscopy, needle biopsy, mediastinotomy, EUS/EBUS biopsy)	
Stage IIIB (not T1-3/N3)	PET/CT scan (if not previously done)<br>Brain MRI<br>Pathologic confirmation (mediastinoscopy, supraclavicular lymph node biopsy; thoracoscopy, needle biopsy, mediastinotomy, EUS/EBUS biopsy)	
Stage IIINOS	PET/CT scan (if not previously done)<br>Brain MRI<br>Pathologic mediastinal lymph node evaluation (mediastinoscopy, mediastinotomy, EBUS, EUS, and CT-guided biopsy.) OR Pathologic confirmation (mediastinoscopy, supraclavicular lymph node biopsy; thoracoscopy, needle biopsy, mediastinotomy, EUS/EBUS biopsy)	
Stage IV<br>Adenocarcinoma, large cell, NSCLC NOS (i.e. non-squamous)	Any molecular testing	

© 2026 Gaddy JJ et al. *JAMA Network Open*.

eTable 1b. Stage-appropriate treatment based on NCCN guidelines.

	**2013-2017**
Stage I	Surgery or RT
Stage IIA	Surgery or RT
Stage IIB (T3, N0)	If tumor size >7 cm: surgery or RT
Stage IIB (T3 invasion, N0)	If tumor size ≤7 cm: surgery or chemoRT
Stage IIB (all others)	Surgery or RT
Stage IINOS	Surgery, RT, or chemoRT (any stage II treatment)
Stage IIIA (T1–3, N2)	Chemo+surgery or chemoRT
Stage IIIA (T3, N1; T4, N0-1)	Surgery or chemoRT
Stage IIIB	ChemoRT
Stage IIINOS	Surgery, chemo, or chemoRT (any stage III treatment)
All Stage IV	Surgery, RT, or chemo

Abbreviations: NCCN, National Comprehensive Cancer Network; PFT, Pulmonary Function Tests; FDG PET/CT, fluorodeoxyglucose Positron Emission Tomography/Computed Tomography; EBUS, Endobronchial ultrasound; EUS, Endoscopic Ultrasound; CT, Computed Tomography; MRI, Magnetic resonance imaging

© 2026 Gaddy JJ et al. *JAMA Network Open*.

eTable 2. County-Level Deprivation and Structural Racism Indices Comparison

County-Level Deprivation (SREI)	County-Level Structural Racism (CSR)
Nine social driver domains:<br><br>1. *Built environment:* Building vacancy rate, mobile homes, no internet access, cancer risk, low food access for SNAP recipients<br><br>2. *Criminal Justice:* Pretrial jail rate (county), total jail rate (county), law enforcement personnel per capital (municipality)<br><br>3. *Education:* Bachelor's degree or higher, high school diploma, per pupil spending (school district)<br><br>4. *Employment:* Unemployed, white-collar occupation, retail job availability<br><br>5. *Housing:* Housing units without telephone, housing units without plumbing, crowding, group quarters, foreclosure risk, eviction risk<br><br>6. *Income and poverty:* Below 100% FPL, below 200% FPL, public assistance, family income, per capita income, supplemental poverty measure<br><br>7. *Social cohesion:* Changed addresses in last year, single-parent households, income gap, residential segregation<br><br>8. *Transportation:* Carpooled to work, no access to a motor vehicle, took public transit to work, biked to work, walked to work, transportation cost burden, median-income family<br><br>9. *Wealth:* Aggregate home value, median real estate taxes paid, median home value, median gross rent, median monthly mortgage, owner-occupied	Five domains:<br><br>1. *Criminal Justice:* (Incarceration ratio) the ratio of non-Hispanic white to black county jail incarceration<br><br>2. *Education:* (High-school graduation ratio) non-Hispanic white to black high school graduation rates<br><br>3. *Employment:* (Poverty ratio) non-Hispanic white to black poverty proportions<br><br>4. *Housing:* A dissimilarity index, which indicates the proportion of the black population that would have to relocate to achieve even distribution<br><br>5. *Health Care:* Diabetes prevention ratio, which indicates the ratio of white non-Hispanic diabetics receiving appropriate A1C monitoring to black diabetics receiving appropriate A1C monitoring
**Scoring System**	
• Mean=0, standard deviation=1<br>• Negative scores: areas richer in resources (i.e., less deprivation)<br>• Positive scores: areas poorer in resources (i.e., more deprivation)	• Mean=0, standard deviation=1<br>• Negative scores: less structural racism<br>• Positive scores: more structural racism

Abbreviations: SREI, Structural Racism Effect Index; CSR: County Structural Racism

© 2026 Gaddy JJ et al. *JAMA Network Open*.

eTable 3. Distribution of Patient Race and Outcomes Across Quintiles of Structural Racism Indices

	SREI (Deprivation Measure)						CSR (Dissimilarity Measure)					
	First Quintile Least Deprivation (N=11,020)	Second Quintile (N=10,804)	Third Quintile (N=11,276)	Fourth Quintile (N=10,280)	Fifth Quintile Most Deprivation (N=10,964)	P-Value[a]	First Quintile Least Structural Racism (N=10,886)	Second Quintile (N=10,867)	Third Quintile (N=10,841)	Fourth Quintile (N=10,139)	Fifth Quintile Most Structural Racism (N=11,611)	P-Value[a]
	N (%)						N (%)					
**Race**[b]						<0.001						<0.001
Non-Hispanic Black	701 (6.4)	552 (5.1)	1,038 (9.2)	1,615 (15.7)	1,682 (15.3)		894 (8.2)	816 (7.5)	750 (6.9)	894 (8.8)	2,234 (19.2)	
Non-Hispanic White	10,319 (93.6)	10,252 (94.9)	10,238 (90.8)	8665 (84.3)	9282 (84.7)		9,992 (91.8)	10,051 (92.5)	10,091 (93.1)	9,245 (91.2)	9,377 (80.8)	
**Localized Stage at Diagnosis**[c] (N = 20,443)	4,400 (21.5)	4,181 (20.5)	4,253 (20.8)	3,732 (18.3)	3,877 (19.0)	<0.001	3,949 (19.3)	4,114 (20.1)	4,126 (20.2)	4,021 (19.7)	4,233 (20.7)	<0.001
**Stage-Appropriate Evaluation and Treatment**[c] (N = 14,807)	3,395 (22.9)	3,292 (22.2)	3,068 (20.7)	2,604 (17.6)	2,448 (16.5)	<0.001	2,681 (18.1)	2,876 (19.4)	3,063 (20.7)	2,991 (20.2)	3,196 (21.6)	<0.001
**Two-Year Survival**[c] (N = 19,469)	4,467 (22.9)	4,149 (21.3)	4,116 (21.1)	3,439 (17.7)	3,298 (16.9)	<0.001	3,546 (18.2)	3,871 (19.9)	3,968 (20.4)	3,896 (20.0)	4,188 (21.5)	<0.001

[a] Bivariate association across the specified structural racism quintiles (Chi-square test).
[b] Percentage calculations are based on column percentages, where the total represents each deprivation/structural racism quintile.
[c] Percentage calculation as based on row percentages, where the total represents the number of patients with the outcome.
SREI: Structural Racism Effect Index; CSR: County Structural Racism

© 2026 Gaddy JJ et al. *JAMA Network Open*.

eTable 4. Association Between Covariates and Outcomes (Bivariate Analysis)

	Localized Stage at Diagnosis		Stage Appropriate Evaluation & Treatment		Two-Year Survival	
	Odds Ratio (95% Confidence Interval)	P-Value[a]	Odds Ratio (95% Confidence Interval)	P-Value[a]	Odds Ratio (95% Confidence Interval)	P-Value[a]
SREI		<0.001		<0.001		<0.001
First	Ref		Ref		Ref	
Second	0.97 (0.88, 1.07)		0.94 (0.82, 1.08)		0.88 (0.80, 0.98)	
Third	0.98 (0.89, 1.09)		0.91 (0.79, 1.05)		0.86 (0.77, 0.96)	
Fourth	0.92 (0.84, 1.01)		0.78 (0.69, 0.88)		0.74 (0.67, 0.81)	
Fifth	0.84 (0.77, 0.91)		0.66 (0.59, 0.74)		0.62 (0.57, 0.68)	
CSR		0.03		0.34		0.002
First	Ref		Ref		Ref	
Second	1.02 (0.94, 1.11)		1.01 (0.89, 1.14)		1.02 (0.92, 1.13)	
Third	0.99 (0.92, 1.08)		1.05 (0.93, 1.19)		1.11 (1.00 1.22)	
Fourth	1.13 (1.03, 1.23)		1.15 (1.00, 1.33)		1.26 (1.12, 1.42)	
Fifth	0.96 (0.88, 1.06)		1.07 (0.92, 1.25)		1.13 (1.00 1.29)	
Age Groups		<0.001		<0.001		<0.001
67-69	Ref		Ref		Ref	
70-74	1.08 (1.01, 1.14)		1.06 (1.00, 1.13)		0.93 (0.88, 0.98)	
75-79	1.07 (1.01, 1.14)		0.97 (0.91, 1.04)		0.77 (0.73, 0.82)	
80-84	0.96 (0.90, 1.02)		0.77 (0.72, 0.83)		0.56 (0.53, 0.60)	
85-94	0.77 (0.72, 0.82)		0.49 (0.45, 0.52)		0.32 (0.30, 0.34)	
Race		<0.001		<0.001		<0.001
NH White	Ref		Ref		Ref	
NH Black	0.73 (0.69, 0.78)		0.69 (0.64, 0.74)		0.74 (0.69, 0.78)	
Sex		<0.001		<0.001		<0.001
Female	1.19 (1.15, 1.23)		1.12 (1.08, 1.16)		1.41 (1.37, 1.47)	
Married	1.09 (1.05, 1.13)	<0.001	1.34 (1.29, 1.40)	<0.001	1.39 (1.34, 1.44)	<0.001
Unknown/Missing	1.24 (1.14, 1.36)		1.09 (0.99, 1.20)		1.31 (1.21, 1.42)	
Dual Eligibility for Medicaid	0.82 (0.78, 0.86)	<0.001	0.62 (0.59, 0.66)	<0.001	0.57 (0.54, 0.60)	<0.001
Year of Diagnosis		<0.001		<0.001		<0.001
2014	0.99 (0.93, 1.05)		1.13 (1.05, 1.22)		1.12 (1.05, 1.19)	
2015	1.03 (0.96, 1.10)		1.21 (1.12, 1.30)		1.19 (1.11, 1.27)	
2016	1.05 (0.99, 1.13)		1.34 (1.25, 1.45)		1.26 (1.18, 1.35)	
2017	1.14 (1.06, 1.21)		1.94 (1.80, 2.09)		1.41 (1.32, 1.51)	
2018	1.21 (1.13, 1.29)		2.22 (2.06, 2.40)		1.45 (1.35, 1.55)	
2019	1.27 (1.18, 1.36)		2.41 (2.24, 2.60)		2.03 (1.90, 2.18)	
Elixhauser Comorbidity Conditions		<0.001		<0.001		<0.001
0	Ref		Ref		Ref	
1-2	1.44 (1.38, 1.52)		1.21 (1.15, 1.27)		1.12 (1.07, 1.17)	
≥3	1.67 (1.59, 1.75)		1.01 (0.96, 1.06)		0.80 (0.76, 0.83)	
Receipt of Flu Shot	1.40 (1.35, 1.46)	<0.001	1.50 (1.43, 1.56)		1.37 (1.32, 1.42)	<0.001
Prior Hospitalizations	1.17 (1.12, 1.22)	<0.001	0.81 (0.78, 0.85)	<0.001	0.70 (0.67, 0.73)	<0.001
PCP Visit	1.46 (1.39, 1.53)	<0.001	2.12 (1.99, 2.25)	<0.001	1.57 (1.50, 1.65)	<0.001
Frailty		<0.001		<0.001		<0.001
Not frail	Ref		Ref		Ref	
Pre-frail	1.21 (1.09, 1.34)		0.88 (0.79, 0.98)		0.79 (0.72, 0.88)	
Mildly frail	1.53 (1.38, 1.71)		0.75 (0.67, 0.84)		0.57 (0.52, 0.64)	
Moderately frail	1.34 (1.19, 1.52)		0.48 (0.42, 0.55)		0.31 (0.27, 0.35)	
Severely frail	1.27 (1.06, 1.52)		0.35 (0.28, 0.44)		0.20 (0.16, 0.24)	

[a] Bivariate analysis with independent factors for each respective outcome.
SREI: Structural Racism Effect Index; CSR: County Structural Racism
Factors that are components of the structural racism indices or directly related to the outcome were excluded from bivariate analyses.

© 2026 Gaddy JJ et al. *JAMA Network Open*.

© 2026 Gaddy JJ et al. *JAMA Network Open*.

eTable 5. Association Between Localized Stage at Diagnosis and County-Level Structural Racism (n = 50,397)

	SREI (Deprivation Measure)[a]		P-Value[a]	CSR (Dissimilarity Measure)[b]		P-Value[b]
	Odds Ratio	95% Confidence Interval		Odds Ratio	95% Confidence Interval	
Race			<0.001			<0.001
Non-Hispanic White	Ref			Ref		
Non-Hispanic Black	0.73	(0.68, 0.78)		0.74	(0.70, 0.79)	
Structural Racism Index			0.02			0.01
First (least structural racism)	Ref			Ref		
Second	0.91	(0.79, 1.06)		1.02	(0.90, 1.15)	
Third	0.92	(0.8, 1.05)		1.01	(0.89, 1.14)	
Fourth	0.84	(0.74, 0.96)		1.21	(1.07, 1.36)	
Fifth (most structural racism)	0.84	(0.74, 0.94)		0.96	(0.86, 1.08)	
Structural Racism * Race Interaction			0.25			0.01
Age Group			<0.001			<0.001
67-69	Ref			Ref		
70-74	1.03	(0.97, 1.10)		1.03	(0.97, 1.10)	
75-79	0.99	(0.93, 1.05)		0.99	(0.93, 1.06)	
80-84	0.87	(0.82, 0.93)		0.87	(0.82, 0.93)	
85-94	0.68	(0.64, 0.73)		0.68	(0.64, 0.73)	
Sex			<0.001			<0.001
Male	Ref			Ref		
Female	1.23	(1.19, 1.28)		1.24	(1.19, 1.29)	
Marital Status			<0.001			<0.001
Not Married	Ref			Ref		
Married	1.12	(1.08, 1.16)		1.12	(1.08, 1.16)	
Unknown/Missing	1.26	(1.15, 1.37)		1.25	(1.15, 1.37)	
Diagnosis Year			<0.001			<0.001
2013	Ref			Ref		
2014	0.98	(0.91, 1.04)		0.98	(0.91, 1.04)	
2015	1.02	(0.96, 1.09)		1.03	(0.96, 1.09)	
2016	1.05	(0.99, 1.12)		1.05	(0.99, 1.12)	
2017	1.13	(1.06, 1.21)		1.13	(1.06, 1.21)	
2018	1.18	(1.11, 1.27)		1.19	(1.10, 1.27)	
2019	1.23	(1.15, 1.32)		1.23	(1.15, 1.32)	
Elixhauser Comorbidity Conditions			<0.001			<0.001
0	Ref			Ref		
1-2	1.45	(1.38, 1.52)		1.45	(1.38, 1.52)	
≥3	1.70	(1.61, 1.80)		1.70	(1.61, 1.80)	
Prior Hospitalizations	1.05	(1.00, 1.10)	0.04	1.05	(1.00, 1.10)	0.04
Frailty			<0.001			<0.001
Not frail	Ref			Ref		
Pre-frail	1.03	(0.93, 1.15)		1.03	(0.92, 1.14)	
Mildly frail	1.12	(1.00, 1.26)		1.11	(0.99, 1.25)	
Moderately frail	0.93	(0.81, 1.07)		0.92	(0.81, 1.06)	
Severely frail	0.86	(0.71, 1.05)		0.85	(0.70, 1.04)	

[a] Multivariable hierarchical logistic model that includes the interaction term (SREI*race) and all additional predictors that demonstrated statistically significant association with the outcome in bivariate analysis.
[b] Multivariable hierarchical logistic model that includes the interaction term (CSR*race) and all additional predictors that demonstrated statistically significant association with the outcome in bivariate analysis.
SREI: Structural Racism Effect Index; CSR: County Structural Racism

© 2026 Gaddy JJ et al. *JAMA Network Open*.

eTable 6. Association Between Appropriate Evaluation and Treatment and County-Level Structural Racism (n = 48,331)

	SREI (Deprivation Measure)[a]		P-Value[a]	CSR (Dissimilarity Measure)[b]		P-Value[b]
	Odds Ratio	95% Confidence Interval		Odds Ratio	95% Confidence Interval	
Race			<0.001			<0.001
Non-Hispanic White	Ref			Ref		
Non-Hispanic Black	0.71	(0.65,0.76)		0.71	(0.66, 0.77)	
Structural Racism Index			<0.001			0.47
First (least structural racism)	Ref			Ref		
Second	0.95	(0.78, 1.15)		0.98	(0.83, 1.15)	
Third	0.87	(0.73, 1.04)		0.95	(0.81, 1.12)	
Fourth	0.80	(0.68, 0.95)		1.10	(0.92, 1.30)	
Fifth (most structural racism)	0.69	(0.59, 0.80)		1.09	(0.92, 1.30)	
Structural Racism * Race Interaction			0.71			0.12
Age Group			<0.001			<0.001
67-69	Ref			Ref		
70-74	1.05	(0.98, 1.12)		1.05	(0.98, 1.12)	
75-79	0.96	(0.89, 1.02)		0.96	(0.90, 1.02)	
80-84	0.77	(0.72, 0.83)		0.78	(0.72, 0.83)	
85-94	0.49	(0.45, 0.53)		0.49	(0.46, 0.53)	
Sex			<0.001			<0.001
Male	Ref			Ref		
Female	1.24	(1.19, 1.29)		1.24	(1.19, 1.30)	
Marital Status			<0.001			<0.001
Not Married	Ref			Ref		
Married	1.30	(1.25, 1.36)		1.30	(1.25, 1.36)	
Unknown/Missing	1.06	(0.96, 1.16)		1.05	(0.95, 1.16)	
Diagnosis Year			<0.001			<0.001
2013	Ref			Ref		
2014	1.11	(1.03, 1.20)		1.11	(1.03, 1.20)	
2015	1.21	(1.12, 1.31)		1.21	(1.12, 1.31)	
2016	1.35	(1.25, 1.45)		1.35	(1.25, 1.45)	
2017	1.95	(1.81, 2.10)		1.96	(1.82, 2.11)	
2018	2.24	(2.08, 2.42)		2.25	(2.08, 2.43)	
2019	2.41	(2.23, 2.60)		2.41	(2.23, 2.60)	
Elixhauser Comorbidity Conditions			<0.001			<0.001
0	Ref			Ref		
1-2	1.35	(1.28,1.42)		1.34	(1.27, 1.42)	
≥3	1.32	(1.24, 1.40)		1.32	(1.24, 1.40)	
Prior Hospitalizations	0.98	(0.93, 1.03)	0.42	0.98	(0.93, 1.03)	0.41
Frailty			<0.001			<0.001
Not frail	Ref			Ref		
Pre-frail	0.83	(0.74, 0.93)		0.83	(0.74, 0.93)	
Mildly frail	0.71	(0.63, 0.80)		0.71	(0.63, 0.80)	
Moderately frail	0.47	(0.40, 0.54)		0.47	(0.40, 0.54)	
Severely frail	0.35	(0.28, 0.45)		0.35	(0.28, 0.45)	

[a] Multivariable hierarchical logistic model that includes the interaction term (SREI*race) and all additional predictors that demonstrated statistically significant association with the outcome in bivariate analysis.
[b] Multivariable hierarchical logistic model that includes the interaction term (CSR*race) and all additional predictors that demonstrated statistically significant association with the outcome in bivariate analysis.
SREI: Structural Racism Effect Index; CSR: County Structural Racism

© 2026 Gaddy JJ et al. *JAMA Network Open*.

eTable 7. Association Between 2-Year Survival and County-Level Structural Racism (N = 54,344)

	SREI (Deprivation Measure)[a]		P-Value[a]	CSR (Dissimilarity Measure)[b]		P-Value[b]
	Odds Ratio	95% Confidence Interval		Odds Ratio	95% Confidence Interval	
Race			<0.001			<0.001
Non-Hispanic White	Ref			Ref		
Non-Hispanic Black	0.76	(0.70, 0.81)		0.78	(0.73, 0.84)	
Structural Racism Index			<0.001			0.007
First (least structural racism)	Ref			Ref		
Second	0.86	(0.73, 1.01)		1.01	(0.88, 1.16)	
Third	0.81	(0.70, 0.94)		1.09	(0.95, 1.25)	
Fourth	0.74	(0.65, 0.85)		1.29	(1.12, 1.49)	
Fifth (most structural racism)	0.66	(0.58, 0.75)		1.13	(0.98, 1.30)	
Structural Racism * Race Interaction			0.20			0.002
Age Group			<0.001			<0.001
67-69	Ref			Ref		
70-74	0.92	(0.87, 0.98)		0.92	(0.87, 0.98)	
75-79	0.78	(0.73, 0.83)		0.78	(0.73, 0.83)	
80-84	0.58	(0.54, 0.62)		0.58	(0.54, 0.62)	
85-94	0.34	(0.31, 0.36)		0.34	(0.31, 0.36)	
Sex			<0.001			<0.001
Male	Ref			Ref		
Female	1.68	(1.61, 1.74)		1.69	(1.62, 1.75)	
Marital Status			<0.001			<0.001
Not Married	Ref			Ref		
Married	1.40	(1.35, 1.46)		1.40	(1.35, 1.46)	
Unknown/Missing	1.30	(1.20, 1.42)		1.30	(1.19, 1.42)	
Diagnosis Year			<0.001			<0.001
2013	Ref			Ref		
2014	1.09	(1.02, 1.17)		1.09	(1.02, 1.17)	
2015	1.19	(1.11, 1.27)		1.19	(1.11, 1.27)	
2016	1.26	(1.18, 1.35)		1.26	(1.18, 1.35)	
2017	1.41	(1.32, 1.51)		1.41	(1.32, 1.51)	
2018	1.45	(1.35, 1.56)		1.45	(1.35, 1.56)	
2019	1.65	(1.54, 1.76)		1.65	(1.54, 1.76)	
Elixhauser Comorbidity Conditions			<0.001			<0.001
0	Ref			Ref		
1-2	1.30	(1.24, 1.37)		1.30	(1.24, 1.37)	
≥3	1.23	(1.16, 1.30)		1.23	(1.16, 1.30)	
Prior Hospitalizations	0.96	(0.91, 1.01)	0.11	0.96	(0.91, 1.01)	0.11
Frailty			<0.001			<0.001
Not frail	Ref			Ref		
Pre-frail	0.76	(0.68, 0.84)		0.76	(0.68, 0.84)	
Mildly frail	0.57	(0.51, 0.63)		0.56	(0.50, 0.63)	
Moderately frail	0.31	(0.27, 0.36)		0.31	(0.27, 0.36)	
Severely frail	0.20	(0.16, 0.25)		0.20	(0.16, 0.25)	

[a] Multivariable hierarchical logistic model that includes the interaction term (SREI*race) and all additional predictors that demonstrated statistically significant association with the outcome in bivariate analysis.
[b] Multivariable hierarchical logistic model that includes the interaction term (CSR*race) and all additional predictors that demonstrated statistically significant association with the outcome in bivariate analysis.

© 2026 Gaddy JJ et al. *JAMA Network Open*.

SREI: Structural Racism Effect Index; CSR: County Structural Racism

© 2026 Gaddy JJ et al. *JAMA Network Open*.
