# Supplementary material for: County-Level Structural Racism Indices and Racial Disparities in Lung Cancer Care
Source: JAMA Netw Open. 2026 May 20;9(5):e2613919. doi: 10.1001/jamanetworkopen.2026.13919 (PMC13191378; doi:10.1001/jamanetworkopen.2026.13919)
Supplement: Supplement 2. — Data Sharing Statement [file jamanetwopen-e2613919-s002.pdf]

# Data Sharing Statement

Gaddy. County-Level Structural Racism Indices and Racial Disparities in Lung Cancer Care. *JAMA Netw Open*. Published May 20, 2026. doi:10.1001/jamanetworkopen.2026.13919

**Data**
**Data available:** No

**Additional Information**
**Explanation for why data not available:** The datasets used to conduct this study are available upon approval of a research protocol from the National Cancer Institute. Instructions for obtaining these data are available at https://healthcaredelivery.cancer.gov/seermedicare/obtain. The collection of the California cancer incidence data used in this study was supported by the California Department of Public Health as part of the statewide cancer reporting program mandated by California Health and Safety Code Section 103885; the National Cancer Institute's Surveillance, Epidemiology and End Results Program under contract N01-PC-35136 awarded to the Northern California Cancer Center, contract N01-PC-35139 awarded to the University of Southern California, and contract N02-PC-15105 awarded to the Public Health Institute; and the Centers for Disease Control and Prevention's National Program of Cancer Registries, under agreement #U55/CCR921930-02 awarded to the Public Health Institute.
